# Structural Analysis of the C-Terminal Region (Modules 18–20) of Complement Regulator Factor H (FH)

**DOI:** 10.1371/journal.pone.0032187

**Published:** 2012-02-28

**Authors:** Hugh P. Morgan, Haydyn D. T. Mertens, Mara Guariento, Christoph Q. Schmidt, Dinesh C. Soares, Dmitri I. Svergun, Andrew P. Herbert, Paul N. Barlow, Jonathan P. Hannan

**Affiliations:** 1 Institute of Structural and Molecular Biology, School of Biological Sciences, King's Buildings, University of Edinburgh, Edinburgh, United Kingdom; 2 European Molecular Biology Laboratory, Hamburg Outstation, Hamburg, Germany; 3 Edinburgh Biomolecular NMR Unit, EaStCHEM, School of Chemistry, University of Edinburgh, Edinburgh, United Kingdom; 4 Centre for Molecular Medicine, Institute of Genetics and Molecular Medicine, The University of Edinburgh, Western General Hospital, Edinburgh, United Kingdom; Griffith University, Australia

## Abstract

Factor H (FH) is a soluble regulator of the human complement system affording protection to host tissues. It selectively inhibits amplification of C3b, the activation-specific fragment of the abundant complement component C3, in fluid phase and on self-surfaces and accelerates the decay of the alternative pathway C3 convertase, C3bBb. We have determined the crystal structure of the three carboxyl-terminal complement control protein (CCP) modules of FH (FH18–20) that bind to C3b, and which additionally recognize polyanionic markers specific to self-surfaces. These CCPs harbour nearly 30 disease-linked missense mutations. We have also deployed small-angle X-ray scattering (SAXS) to investigate FH18–20 flexibility in solution using FH18–20 and FH19–20 constructs. In the crystal lattice FH18–20 adopts a “J”-shape: A ∼122-degree tilt between the structurally highly similar modules 18 and 19 precedes an extended, linear arrangement of modules 19 and 20 as observed in previously determined structures of these two modules alone. However, under solution conditions FH18–20 adopts multiple conformations mediated by flexibility between CCPs 18 and 19. We also pinpoint the locations of disease-associated missense mutations on the module 18 surface and discuss our data in the context of the C3b:FH interaction.

## Introduction

Complement factor H (FH), a 155-kDa soluble glycoprotein abundant in human plasma, is an important regulator of the complement system, the chief molecular component of innate immunity. FH is a member of the regulators of complement activation (RCA) family (reviewed in [1]) that are characterized by possession of repeating compact domains, known as complement control protein modules (CCPs), sushi domains or short consensus repeats [2], [3]. In FH twenty CCPs are arranged in a tandem manner [4].

Human FH binds to, and regulates levels of, the first activation-specific cleavage product of complement component C3, C3b. Without regulation, C3b self-promulgates via formation of the complex, C3bBb, which proteolyses C3, producing more C3b. C3b can attach indiscriminately to surfaces *via* a thioester-containing domain (TED), and is an opsonin. It also triggers a proteolytic cascade terminating in self-assembly of cytolytic pores. FH is a cofactor for proteolysis of C3b to iC3b that no longer participates in the complement cascade but remains opsonic and is a ligand for receptors on B-cells and phagocytes. FH also competes with factor B for binding to C3b, and it accelerates the irreversible decay of C3bBb [5]–[7]. Furthermore, FH recognizes polyanionic markers, such as glycosaminoglycans (GAGs) that are common on self-surfaces but rare on pathogen surfaces [8]–[10]. This dual C3b and polyanion recognition allows FH to regulate complement activation effectively on self-surfaces but not on foreign ones [10].

The four FH amino-terminal CCPs (1–4) perform co-factor and C3bBb decay-accelerating activities, while the two carboxyl-terminal CCPs (19–20) also bind C3b but additionally bind to GAGs [11]–[14]. The crystal structures of a FH1–4:C3b complex [15], confirmed separately by fluorescence resonance energy transfer-derived data [16], and of FH19–20 complexed with C3d (a 35-kDa C3 opsonic proteolytic fragment equivalent to the TED in C3b) have previously been determined [17], [18]. Principal GAG (and sialic acid)-binding regions occupy CCPs 6–8 and CCP 20 [13], [17], [19]–[24]. CCPs 19–20 recognise a composite binding site consisting of surface-tethered C3d (TED) and nearby GAGs, and are thus crucial for ensuring FH acts most effectively at self-surfaces [18]. In current models, the 20 FH CCPs, connected by linkers of three to eight residues, adopt a bent-back conformation with varying degrees of flexibility between modules [18], [25]–[29]. The two FH termini simultaneously engage proximal binding sites on a common surface-tethered C3b molecule; the intervening 14 modules loop out such that CCPs 6–8 approach the surface, reinforcing the GAG-recognition properties of CCP 20 [17], [18].

Given its key role in host cell protection it is unsurprising that FH sequence variations associate with diseases including membranoproliferative glomerulonephritis type II, age-related macular degeneration (AMD) and atypical haemolytic uraemic syndrome (aHUS) (reviewed in [30], and [31]). Interestingly, the majority of disease-associated missense mutations are clustered in CCPs 18–20 [31]–[36].

In this study we describe the crystal structure of the three carboxyl-terminal CCP modules of human FH (CCPs 18–20) at a resolution of 1.8 Å together with analysis of this region in solution by small angle X-ray scattering (SAXS). This new structural information extends current knowledge based on the CCPs 19–20 structures and provides a more robust structural context for discussion of disease-linked mutations.

## Methods

### Expression and Production of Recombinant FH18–20 & FH19–20

Recombinant versions of FH18–20 and FH19–20 comprising residues 1046–1231 and residues 1107–1231 (UniProt accession number: P08603), respectively, were expressed and purified as previously described [18], [37]. Recombinant FH18–20 contained an amino-terminal two-residue cloning artefact (Ala-Gly), while FH19–20 contained an amino-terminal four-residue cloning artefact (Glu-Ala-Glu-Phe). The FH18–20 construct which contains a single N-glycosylation consensus sequence (Asn1095-Trp1096-Thr1097) was deglycosylated using endoglycosidase H_f_ (New England Biolabs), according to the manufacturer's instructions, prior to use. For crystallization or small angle X-ray scattering (SAXS) studies, FH18–20 and FH19–20 were concentrated by centrifugation using a Vivaspin 20 (Millipore) concentration device (10 KDa molecular weight cut-off) at 4000× g, 20°C in phosphate-buffered saline (PBS), pH 7.4 (containing 137 mM NaCl, 8.1 mM Na_2_HPO_4_, 2.7 mM KCl, 1.5 mM KH_2_PO_4_).

### FH18–20 Crystallization and Data Collection

Crystals of FH18–20 were grown at 17°C by vapor diffusion from hanging drops. Drops contained 1 µl of protein solution (16.8 mg/ml) in PBS with an equal volume of well solution (0.1 M sodium malonate, pH 4.0, 12% w/v polyethylene glycol 3350). Crystals grew within forty-eight hours. Crystals were flash frozen in liquid nitrogen after successive soakings in cryoprotectant solutions containing 10% and 25% v/v glycerol. Intensity data were collected (ϕ scans were 1° over 180°) to a resolution of 1.8 Å (the edge of the detector) on beamline I03 at the Diamond Light Source (Oxfordshire, UK). Data were indexed with Mosflm [38], and subsequently merged and scaled with SCALA [39].

### FH18–20 Structure Determination

A previously elucidated structure of FH19–20 (PDB ID: 3OXU/chain F [18]) was used as a search model for molecular replacement using the program PHASER [40]. The resulting model underwent ten cycles of restrained refinement using the program REFMAC [41]. The remaining CCP module (FH18) was built using the PHENIX Autobuild Wizard [42] and the program COOT [43]. This model was subjected to further cycles of restrained refinement and, when appropriate, ligands and water molecules were added to the model using COOT. Disordered regions were carefully modeled into *F_o_−F_c_* electron density and changes in *R/R*_free_ (%) values were used to assess final model quality.

The final structure was composed of one FH18–20 molecule comprising 185 residues (Gly1045-Lys1230), 17 of which exhibit alternate conformations, 170 water molecules, four glycerol molecules and a phosphate ion. No clear electron density was observed for the first or last residues in the recombinant FH18–20 sequence (Ala1044 or Arg1231), while the Thr1184–K1188 region within CCP 20 was disordered; this region was modeled using the NMR-derived FH19–20 structure (PDB ID: 2BZM [37]). The *R/R*_free_ values converged for twenty cycles of *REFMAC* at 18.2% and 22.6%, respectively. Data-reduction and refinement statistics are summarized in Table 1. Figures were generated using the PyMOL Molecular Graphics System (Version 1.3, Schrödinger, LLC).

**Table 1:** Crystallographic data collection and refinement statistics.

Data collection	
Space group	P22_1_2_1_
Cell dimensions	
*a*, *b*, *c* (Å)	45.97, 68.60, 77.48
Resolution (Å)	51.36-1.80
*R*_merge_ (%)	5.9 (30.9)
*I*/σ*I*	13.7 (3.5)
Completeness (%)	99.7 (99.9)
Multiplicity	4.1 (4.1)

Refinement	
No. reflections	22011
*R*_work_/*R*_free_ (%)	18.2/22.6
No. atoms	
Protein	1570
Ligand	28
Water	170
Average protein *B*-factor (Å^2^)	29.5
R.m.s. deviations	
Bond lengths (Å)	0.03
Bond angles (°)	2.05
Ramachandran	
allowed (%)	98.0 (198/202)
generous (%)	100.0 (202/202)
disallowed (%)	0.0 (0/202)

Values in parentheses are for the highest resolution shell. 5% of reflections were used as a test set for the calculation of R_free_.

### Validation and Deposition of FH18–20 Structure

The geometry of the model was assessed using MolProbity [44]. Atomic coordinates and the experimental structure factors for our 1.8 Å structure of FH18–20 have been deposited in the Protein Data Bank with the accession code 3SW0 (PDB ID: 3SW0).

### Small-Angle X-ray Scattering

Synchrotron radiation X-ray scattering data were collected on the X33 beam line of the EMBL (DESY, Hamburg, Germany) using a Pilatus one-megapixel array detector (Dectris, Switzerland) and eight frames of 15-second exposure times. Solutions of FH18–20 and FH19–20 were measured at 20°C in PBS, pH 7.4, 1 mM DL-Dithiothreitol (DTT) at protein concentrations of 1.9, 3.8 and 7.6 mg/ml (for FH18–20) and 2.4, 4.6 and 7.9 mg/ml (for FH19–20). The sample-to-detector distance was 2.7 m, covering a range of momentum transfer 0.07 nm^−1^<*s*<6.0 nm^−1^ (*s* = 4πsinθ/λ, where 2θ is the scattering angle, and λ = 0.15 nm is the X-ray wavelength). Addition of reducing agents, such as DTT, serve as free radical scavengers and can significantly reduce radiation damage to biological samples [45]. Comparison of successive 15-second frames revealed no detectable radiation damage in the presence of DTT. However, in the absence of DTT, significant radiation damage occurred following the second frame. Usage of 1 mM DTT in our hands has been shown to be insufficient to reduce disulphide bonds within CCP-containing constructs (data not shown). Data from the detector were normalized to the transmitted beam intensity, averaged and the scattering of buffer solutions subtracted. Difference curves were scaled for solute concentration. All data manipulations were performed using the *PRIMUS* software package 16 [46].

Fitting of the FH18–20 and FH19–20 (PDB ID: 3OXU [18]) crystal structures to the SAXS data was conducted using the program CRYSOL [47]. CRYSOL calculates the partial scattering amplitudes of proteins from their atomic coordinates, taking into account the hydration layer and excluded solvent volume. Low resolution shape envelopes were determined from the solution scattering data using the program DAMMIF [48] and the most typical model from multiple reconstructions (10) identified using DAMAVER [49]. Resulting bead models were converted to meshed envelopes and visualized using PYMOL (Version 1.3, Schrödinger, LLC). Superposition of available bead models on three-dimensional structures of FH18–20 or FH19–20, as appropriate, were carried out using the program SUPCOMB13 [50]. Rigid body modeling using the program CORAL (Complexes with Random Loops) was also conducted using the FH18–20 crystal structure, constraining either FH modules 18 and 19, or 19 and 20 as fixed, and refining the relative position and orientation of modules 20 or 18, respectively, against the SAXS data [51].

Analysis of inter-domain flexibility in FH18–20 employed the ensemble optimization method (EOM) [52]. This uses a genetic algorithm to select, from a pool of randomly generated models, an ensemble of possible conformations whose combined theoretical scattering profiles best fit the experimental data. The CCP modules of FH18–20 were treated as rigid bodies and the linkers between them represented as flexible chains of dummy residues. For the pool, 10,000 models were generated from the input structures. A final ensemble of 20 conformations was selected by genetic algorithm after 50 cycles.

The discrepancies (χ) between models/ensembles and the experimental data from CRYSOL, DAMMIF, CORAL and EOM are summarised in Table S1. This discrepancy is defined as:
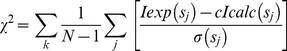
(1)where *N* is the number of experimental points, *I_exp_(s_j_)* and *I_calc_(s_j_)* are the experimental and calculated scattering intensities, *c* is a scaling factor and *σ(s_j_)* is the experimental error at the momentum transfer *s_j_*.

## Results

### Crystal Structure of FH18–20

**Figure 1:**
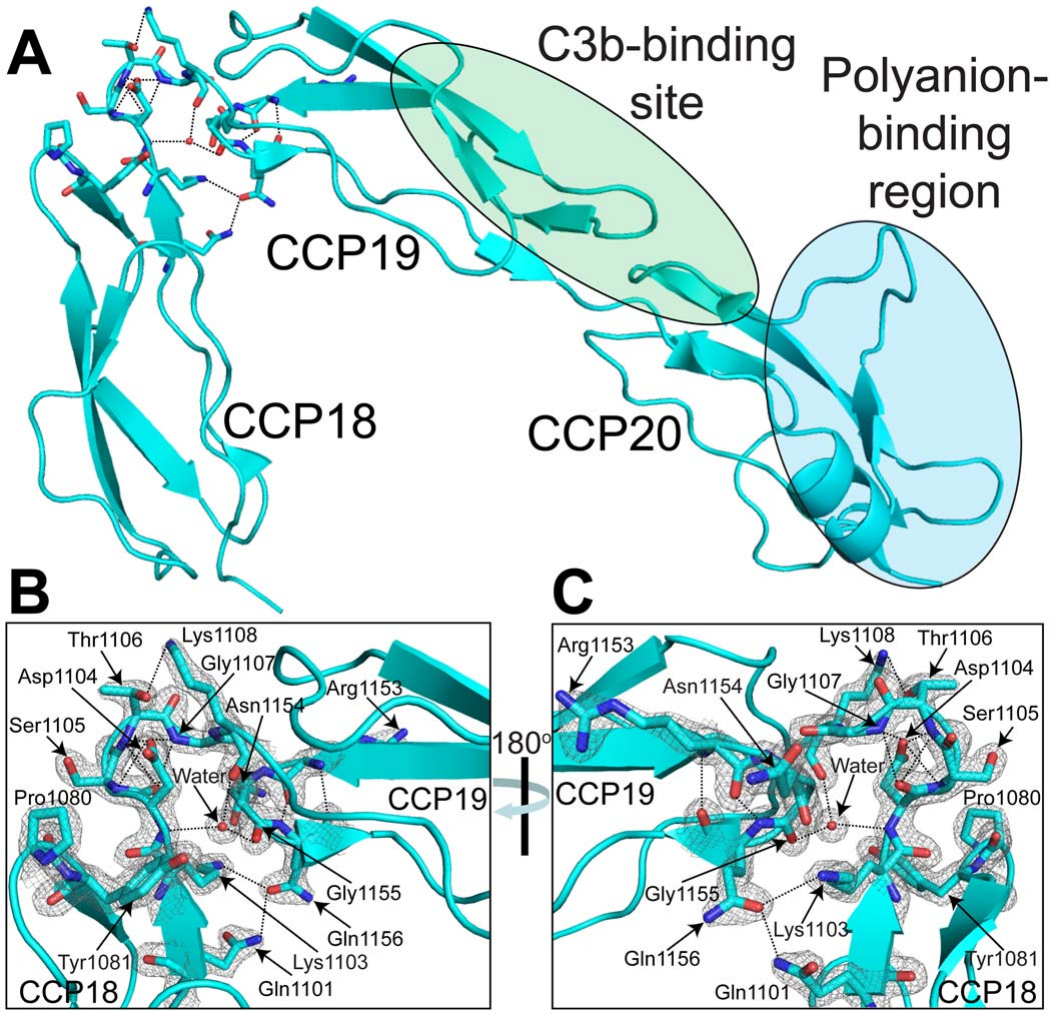
Crystal structure of FH18–20 (PDB ID: 3SW0). A, A Cartoon representation of the three CCP modules is indicated: CCP 18 (residues 1048–1102), CCP 19 (residues 1109–1163), and CCP 20 (residues 1167–1228). Highlighted on the FH18–20 structure are the C3b-binding (green) and polyanion-binding (blue) regions [17], [18], [21]. Residues contributing to the inter-domain packing between CCPs 18 and 19 are shown. B, Close-up of the kink that occurs between modules 18 and 19. The orientation of the FH18–20 molecule is the same as shown in ‘A’. Electron density (2*F*o−*F*c map shown in grey, and contoured at 1.5σ) for residues contributing to the inter-modular packing is shown. Dashed lines represent hydrogen-bonds between amino acid residues or between amino acid residues and water molecules. C, as for ‘B’ except the molecule is rotated about the *y*-axis by 180°.

FH18–20 crystals diffracted to a maximum resolution of 1.8 Å (see Table 1). FH18–20 data were indexed in the space-group P22_1_2_1_, with one monomer in the asymmetric unit. The three CCP modules of FH18–20 form a ‘J’-shaped structure (Figure 1A). CCPs 19 and 20 adopt an extended rod-like conformation in which CCP 20 is approximately aligned with CCP 19, consistent with previous structural studies carried out using wild-type or mutant forms of FH19–20 [17], [18], [22], [37], [53], [54]; CCPs 19–20 of our FH18–20 structure (PDB ID: 3SW0) may be superimposed (residues 1109–1228, back-bone atoms) with a root-mean-square (rms) of 1.21 Å on a crystal structure of wild-type FH19–20 (PDB ID: 3OXU/chain F [18]). Both FH18 and FH19 are typical CCP modules with very similar structures (rms, alpha-carbon atoms, ∼1 Å) in line with their high sequence similarity, while CCP 20 exhibits great structural divergence (rms, alpha-carbon atoms >2 Å to all but one other CCP module) (Table S2) [55]. While the long axes of CCPs 19 and 20 are approximately aligned, with only a ∼32° tilt relative to one another, the long axis of CCP 18 is strongly tilted, by ∼122° with respect to the long axis of CCP 19, and by ∼151° with respect to CCP 20 (Table S3). This distinctive kink in the FH18–20 structure is facilitated by an extensive network of hydrogen bonds (Figure 1). Atomic distances consistent with hydrogen bond formation are observed between Gln1101 (CCP 18)-Gln1156 (CCP 19); Lys1103 (linker)-Asn1154 (CCP 19); and Lys1103 (linker)-Gln1156 (CCP 19). In addition, a single water molecule (B-factor = 25 Å^2^) forms three hydrogen bonds, one with the amide of Asp1104 (linker) and two with the backbone carbonyl oxygen atoms of Lys1108 (linker) and Gly1155 (CCP 19). Further hydrogen bonding involving residues: Asp1104 (linker)-Ser1105 (linker); Asp1104 (linker)-Gly1107 (linker); Thr1106 (linker)-Lys1108 (linker); and Arg1153 (CCP 19)-Gln1156 (CCP 19) (Figure 1B,C) also contribute to the kink between CCPs 18 and 19. This network of hydrogen bonds may also stabilize the observed CCP 18–CCP 19 inter-modular orientation under solution conditions; however, we cannot rule out the possibility that the distinctive kinked structure is induced by crystal contacts, or alternatively, by the low-pH conditions employed to crystallize the molecule. Furthermore, the surface area buried between CCPs 18 and 19 is only ∼400 Å, compared to almost ∼700 Å for CCPs 19 and 20 (Table S4).

### SAXS Analysis of FH18–20 and FH19–20

**Figure 2:**
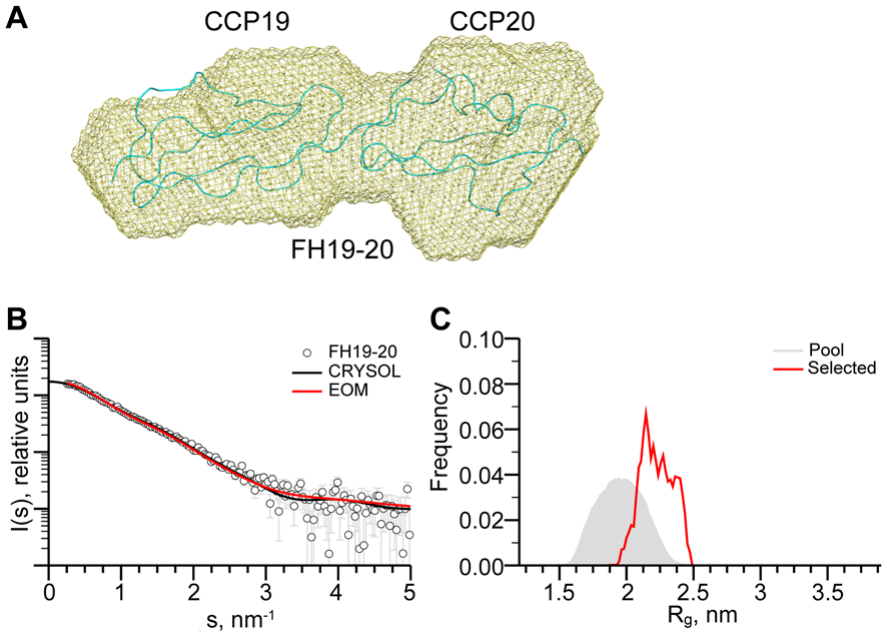
Summary of FH19–20 SAXS data. A, Superposition of the SAXS-derived shape envelope of recombinant FH19–20 (shown in yellow) on the crystal structure of FH19–20 (PDB ID: 3OXU [18]). Shape envelopes were determined using the *ab initio* bead-modelling program DAMMIF [48] and superposition of the FH19–20 envelope on the corresponding crystal structure was carried out utilizing the program, SUPCOMB13 [50]. B, Fit of the X-ray crystal structure of FH19–20 (solid black line) to the SAXS data extrapolated to infinite dilution (black open circles). The fit of the selected ensemble of conformations from EOM is also shown (solid red line). C, The *R_g_* distribution from the ensemble analysis using EOM (pool in grey, selected ensemble in red).

To investigate the conformation adopted by these three C-terminal CCP modules in solution, SAXS data were acquired on samples of both FH18–20 and FH19–20 (Table 2). For the double module, FH19–20, the overall parameters suggest that the sample is predominantly monomeric in solution (Table 2). The MW as estimated from the forward scattering intensity of the merged data extrapolated to infinite dilution, *I(0)* is ∼15 kDa, and along with estimates derived from the hydrated particle volumes and *ab initio* bead modelling, is consistent with monomeric FH19–20 in solution (Figure 2A). Fits of the crystal structure of this FH19–20 construct (PDB ID: 3OXU [18]) to the SAXS data using the program CRYSOL are shown in Figure 2. The structure of FH19–20 provides an excellent fit (χ = 1.4) to the merged scattering data extrapolated to infinite dilution (Figure 2B), supporting the extended structures previously solved by both X-ray crystallography and NMR for this fragment [17], [18], [22], [37], [53], [54]. The presence of potential flexibility in the three-residue linker between CCPs 19 and 20 was investigated using an ensemble-optimization analysis conducted using the program EOM. The *R_g_* distribution from this analysis is characteristic of an extended structure with a small degree of conformational flexibility relative to the pool of random conformers (Figure 2C).

**Table 2:** Overall SAXS parameters.

	Concn mg/ml)	*R_g_^Guinier^* (nm)	*R_g_^GNOM^* (nm)	*D_max_* (nm)	*I(0)*	MW*^SAXS^* (kDa)	Vol*^SAXS^*, nm^3^	Vol*^DAM^*, nm^3^
FH19–20 (14.9 kDa)	2.4	2.3±0.1	2.3±0.1	8.0±0.5	17.4	14±5	17	23±5
	4.6	2.4±0.1	2.4±0.1	8.4±0.5	19.2	16±5	20	26±5
	7.9	2.6±0.1	2.6±0.1	9.0±0.5	21.2	21±5	23	32±5
	mer	2.2±0.1	2.2±0.1	7.4±0.5	18.2	15±5	16±5	22±5
FH18–20 (21.4 kDa)	1.9	3.0±0.1	3.2±0.1	10.6±0.5	21.7	18±5	21±5	29±5
	3.8	3.1±0.1	3.3±0.1	10.9±0.5	22.7	18±5	23±5	32±5
	7.6	3.4±0.1	3.5±0.1	11.5±0.5	26.6	22±5	29±5	39±5
	mer	2.9±0.1	3.2±0.1	10.2±0.5	21.9	22±5	30±5	32±5

*R_g_^Guinier^* and *R_g_^GNOM^* are the experimentally determined radius of gyration as calculated by Guinier analysis [62] and by indirect Fourier transform using the program GNOM, respectively [63]; *D_max_* is the maximum particle dimension; *I(0)* is the forward scattering intensity; *MW^(SAXS)^* is the molecular weight determined by SAXS; Vol*^SAXS^* is the hydrated particle volume of solutes determined from the SAXS patterns; and Vol*^DAM^* is the excluded volume of solutes determined using the *ab initio* modeling program DAMMIF [48]. Data merged and extrapolated to infinite dilution are referred to in the table as “mer”.

**Figure 3:**
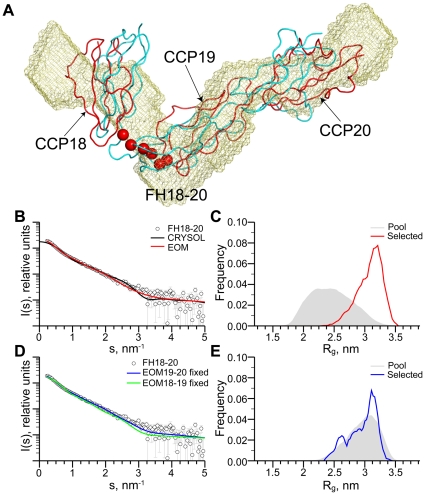
FH18–20 SAXS analysis. A, Superposition of the SAXS-derived shape envelope of recombinant FH18–20 (yellow) on the crystal structure of FH18–20 (indicated in cyan), and also on a CORAL-derived rigid body model of FH18–20 (shown in red) where the orientation of CCPs 19–20 are fixed, and the position of CCP 18 refined against the SAXS data. Flexible linker residues (alpha-carbon atoms) are shown as red spheres. B, Fit of the FH18–20 crystal structure (black line) to the SAXS data using the program CRYSOL, and fit of the selected ensemble of FH18–20 models from the EOM analysis (red line) to the SAXS data. C, *R_g_* distribution from the EOM analysis of FH18–20 with both FH18–19 and FH19–20 linker regions defined as flexible (pool in grey, selected ensemble in red). D, Fits of the selected ensembles from the EOM analysis of FH18–20 to the SAXS data using flexible FH18–19 (blue line) or FH19–20 (green line) linker regions. E, *R_g_* distribution from the EOM analysis for FH18–20 with the FH18–19 linker region defined as flexible (pool in grey, selected ensemble in blue).

For FH18–20, the MW estimate of ∼22 kDa from the merged data extrapolated to infinite dilution agreed with that expected for a monomeric form of this three-module protein. Volume estimates were also consistent with a monomeric state, with volumes of 30 and 32 nm^3^ measured for the *Vol^SAXS^* and *Vol^DAM^* values, respectively (corresponding to estimated MW's of 19±5 kDa and 16±5 kDa). Interestingly, though, the experimentally derived scattering curves did not fit well to data back-calculated from the ‘J’-shaped crystal structure (discrepancy χ = 2.4) (Figure 3); nor did *ab initio* low-resolution shape envelopes generated using the program DAMMIF demonstrate the same acute angle between CCPs 18 and 19 (Figure 3A) [48]. Furthermore, SAXS-based rigid body models of FH18–20 generated using the program CORAL, in which the position and orientation of CCP 18 was refined against the SAXS data, resulted in an average solution conformation which was more extended than the crystal structure (Figure 3A) and which better fit the scattering data (χ = 1.4) (Figure S1). By contrast, refinement of the position and orientation of CCP 20 yielded no improvement in the fit to the SAXS data compared to that of the crystal structure (χ = 2.1) (Figure S1). Overall, these SAXS results suggest FH18–20 has a more extended conformation in solution than that observed in the crystal lattice. The most straightforward explanation is that under the conditions used to collect the SAXS experiments, FH18–20 is more flexible than might be inferred from its crystal structure.

To investigate potential flexibility of the linker regions within CCPs 18–19 and CCPs 19–20, an analysis was carried out with EOM [52]. When all inter-module linkers were defined as flexible the genetic algorithm selected an ensemble of conformations providing a superior fit (χ = 1.2) to the SAXS data, compared to that of the crystal structure (Figure 3B). The *R_g_* distribution of the selected ensemble from this analysis is shifted toward extended structures while the width of the distribution is smaller than that of the pool (Figure 3C), suggesting partial flexibility. To investigate the location of potential flexibility and reduce the number of degrees of freedom of the EOM analysis, two additional runs were conducted. In these, the linker between CCPs 18 and 19 or between 19 and 20 was fixed as in the crystal structure, allowing either CCP 20 or CCP 18, respectively, (*via* the remaining non-fixed linker) to sample conformational space. The SAXS data were fit well by an ensemble in which the 19–20 linker was fixed (χ = 1.3) (Figure 3D), but were fit poorly by the ensemble in which the 18–19 linker was fixed (χ = 2.4). These data, which are entirely consistent with the experiments performed on FH19–20, suggest that the six residue 18–19 linker, but not the three residue 19–20 linker, is significantly flexible. The *R_g_* distribution of the selected ensemble in which the 19–20 linker was fixed coincides well with that of the respective pool, being both broad (and thus considerably flexible) and skewed toward extended structures (Figure 3E).

**Figure 4:**
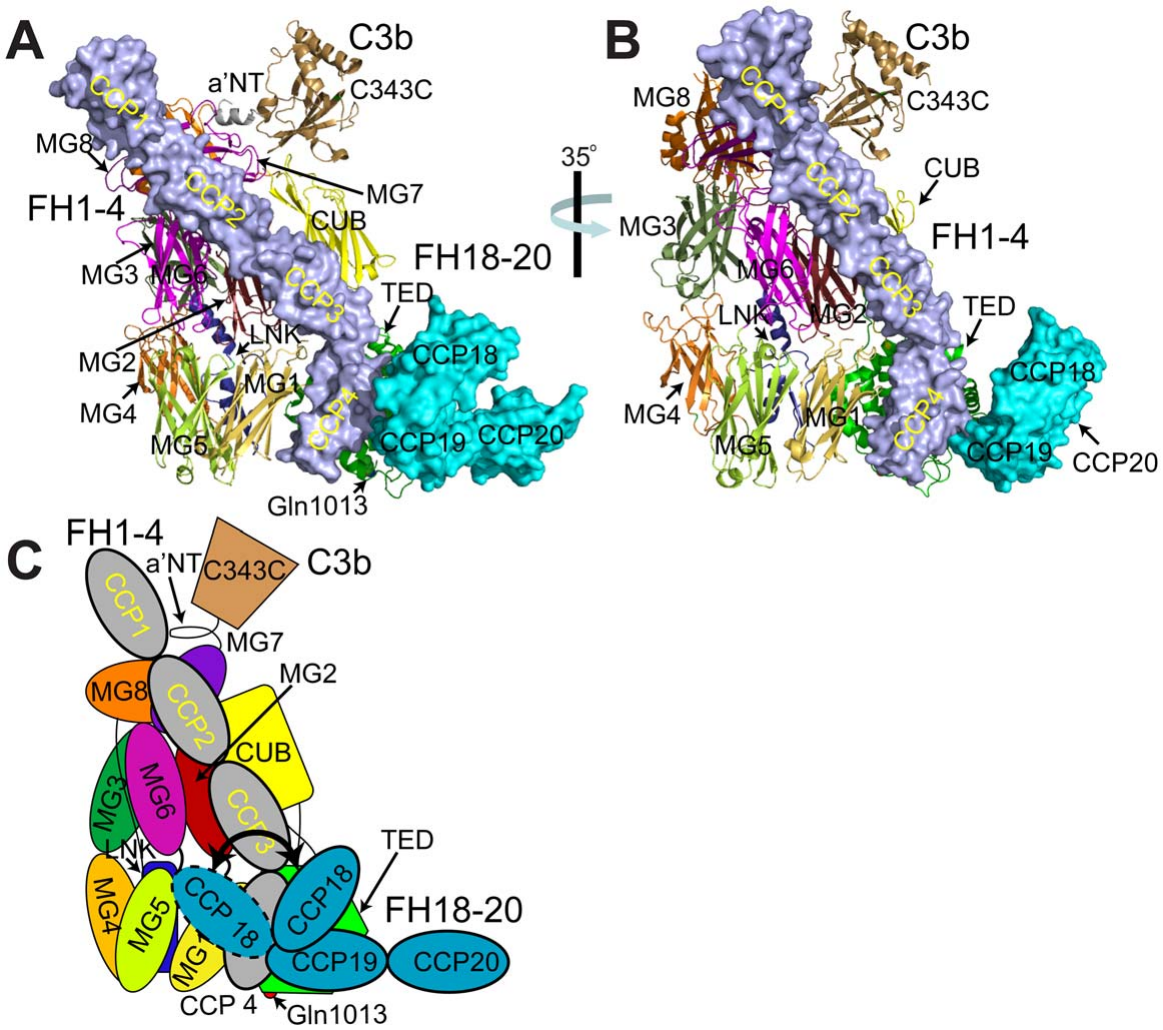
The crystal structure of FH18–20 modeled onto the C3b:FH1–4 complex. A, Superposition of FH18–20 structure (PDB ID: 3SW0) on the previously determined wild-type FH19–20:C3d complex (PDB ID: 3OXU [18]) and the FH1–4:C3b complex (PDB ID: 2WII [15]). Surface representations are shown of FH1–4 (slate) and FH18–20 (cyan). In the cartoon representation of C3b, constituent domains are color-coded with the TED indicated in green. FH19–20 and C3d were employed for alignment purposes only, and are not shown. Also indicated is Gln1013, the site of covalent linkage of C3b to target surfaces. B, As for (A) except the model of the FH1–4:C3b:FH18–20 complex has been rotated about the *y*-axis by 35° demonstrating the path of CCP 18 with respect to the FH1–4:C3b complex. C, Schematic of a FH1–4:C3b:FH18–20 complex demonstrating the inferred flexibility, in solution, of the linker connecting CCPs 18 and 19.

In summary, these data are consistent with a solution of, on average, more extended FH18–20 conformations (compared to the crystal structure) with a rigid 19–20 linker and a highly flexible 18–19 linker. It is possible therefore, that the kinked crystal-derived FH18–20 structure in which CCP 18 folds back towards CCP 19 reflects a snapshot of one of several conformations available to these three modules (Figure 4).

## Discussion

**Figure 5:**
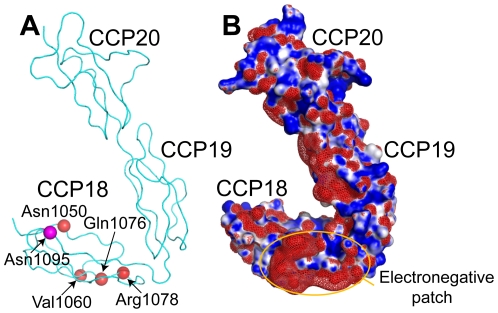
Location of disease-associated missense mutations within CCP 18. A, Shown are the alpha-carbons (red spheres) of residues for which missense mutations associated with aHUS or basal laminar drusen have been reported. Residue numbers are: 1050 (basal laminar drusen variant N1050Y) [31]; 1060 (aHUS-associated variant V1060A) [36]; 1076 (aHUS-associated variant Q1076E) [32], [34]; and 1078 (basal laminar drusen-associated variant R1078S) [31]. Also indicated in magenta is the alpha-carbon of residue 1095, the Asn of the sole N-glycosylation consensus sequence located within FH18–20. B, Electrostatic surface representation of FH18–20. Positively and negatively charged areas are indicated in blue and red, respectively. Also shown as a red mesh is a negative isosurface map contoured at −2 kT/e. This figure was generated using the APBS plug-in for PyMOL [64].

We have extended the structural information available for the key soluble human complement regulator, FH. We elucidated the crystal structure of FH18–20, and have complemented this with the acquisition of solution SAXS data for FH19–20 and FH18–20. These C-terminal CCPs encompass the key self *versus* non-self discriminating region of this protein [10]. While several high-resolution structures of the C3b/GAG-contacting CCPs 19 and 20 were already available, CCP 18 is also of interest since it too is a site of disease-associated mutations (Figure 5) [31], [35], [36]. Moreover, the inter-modular angles between CCPs 18 and 19 are important because they determine the path of the carboxyl-terminal region of the FH molecule as it exits the C3b:FH complex before looping back to re-engage with the same C3b molecule via its amino-terminal CCPs [18].

FH18–20, in the crystal lattice, adopts a distinctive ‘J’-shaped conformation. The short (three-residue) linker between CCPs 19 and 20 (*i.e.* between the last cysteine of CCP 19 and the first of CCP 20) along with numerous inter-modular contacts imposes an approximately linear rod-like structure on this (GAG/C3b-binding) part of the molecule; the longer (six-residue) 18–19 linker permits a sharp kink to form in the molecule, also stabilized by inter-modular and module-to-linker interactions, but with a smaller inter-modular interface than observed between modules 19 and 20. Interestingly, a previously published SAXS-based model of FH 15–19 also contained a kink between CCPs 18 and 19 [18]. On the other hand our SAXS-derived analysis of FH18–20 and FH19–20 in solution revealed the presence of conformational mobility at the 18–19 inter-modular junction, but little flexibility between CCPs 19 and 20. These data are consistent with the low buried surface area which is observed between CCPs 18 and 19 (Table S3). Varying levels of inter-modular flexibility have been noted previously in FH and other RCAs [13], [56]–[58].

Taking these data together, it appears that modules 19 and 20 are rigidly aligned such that their rod-like conformation is independent of the addition of CCP 18 or the presence of ligands. Modules 18 and 19, on the other hand, can adopt a bent-back conformation, supported by numerous non-covalent interactions, that would bring residues on the surface of module 18 in close proximity to C3b within a FH:C3b complex rather than projecting module 18 (and the preceding modules of FH) clear of the complex (Figure 4); but this junction is much less rigid than that between modules 19 and 20. Occupation of a solitary N-glycan site on Asn1095 within CCP 18 (Figure 5) would not preclude this conformational flexibility due to its remoteness from the CCP 18–CCP 19 linker.

The carboxyl-terminal region of FH is a hotspot for disease-associated mutations which have been linked to increased risk for the development of aHUS, early onset drusen (basal laminal drusen) and age-related macular degeneration (AMD) [31]–[36]. To date, at least twenty-eight such missense mutations have been documented in FH18–20, four of which occur in CCP 18: N1050Y [31]; V1060A [36]; Q1076E [32], [34]; and R1078S [31]. All of these substitutions are located on the surface of CCP 18 (Figure 5A) and none is likely to result in significant structural perturbations; two of them (Q1076E and R1078S), however, alter the electrostatic potential of CCP 18 and are, additionally, in close proximity to an electronegative patch on this module (Figure 5B). While direct binding to C3b occurs mainly through CCP 19 and the CCP 19–20 inter-modular junction, residues exposed on CCP 18 could nonetheless play a role in the encounter between the C terminus of FH and C3b and therefore modulate the ability of FH to control C3b amplification on host surfaces. In previous work reversal-of-charge mutations in CCPs 19 and 20 were found to influence affinity of FH19–20 for C3d/C3b even when they did not lie directly in the interface between these two molecules as visualized in the crystal structure of the FH19–20:C3d complex [17], [18], [59], [60]. Such observations were attributed to electrostatic steering. It has also been suggested that some disease-associated mutations in FH19–20 modulate self-association [54]. The wild-type FH18–20 protein is monomeric under the conditions used for SAXS. Mutagenesis combined with binding and biophysical studies would be needed to explore the hypothesis that residues in CCP 18 exert electrostatic steering effects, or that mutations in CCP 18 can influence self-association. Alternatively, interactions with other less well established FH ligands might be directly affected by mutations in CCP 18; the inflammatory biomarker, C-reactive protein, for example, has been reported to bind to a FH16–20 construct [61].

## Supporting Information

Figure S1 **Fit of FH18–20 rigid body models refined against the SAXS data using the program CORAL.** Fits are shown for rigid body models refining only CCP 18 (CCPs 19–20 fixed) or CCP 20 (CCPs 18–19 fixed), as solid blue and dashed red lines, respectively.
(PPT)

Table S1 **Comparison of the discrepancies (χ) for the DAMMIF, CORAL and EOM SAXS-derived analyses.**
(DOC)

Table S2 **Pair-wise CCP module structural comparisons of FH18, FH19 and FH20.** Comparison of CCP module structures of CCP 18, CCP 19 and CCP 20 versus all other individual CCPs of known structure within the complement system based upon alpha-carbon RMSD values using the structural alignment program Combinatorial Extension [22]. For each CCP, inclusive module boundaries were one residue before Cys-I and the third residue after Cys-IV. In cases where structures have been solved by both NMR and X-ray diffraction, the higher resolution X-ray structure was used for comparison. Where both liganded and unliganded structures were available, the highest resolution unliganded X-ray or NMR structure was used. A few residues were missing in the crystal structure of C1r CCP 2, and hence in this case, the structure with the most determined residues was employed for both modules. Colour key used in table: Blue: 0–1.99 Å; Green: 2.00–2.99 Å; Red: 3.00–3.99 Å; Brown: Alignment lengths <40 amino acids. Abbreviations used in Table: C4BPα = C4b-binding protein α-chain; CR = complement receptor; CRRY = rat Complement receptor 1-related protein Y; DAF = decay-accelerating factor; FB = factor B; FH = factor H; MASP1/2 = mannan-binding lectin-associated serine proteases 1/2; MCP = membrane cofactor protein; VCP = Vaccinia virus complement control protein. Some residues were not present (solved) in the electron density map for the C1r CCP 2 module crystal structure, and this explains the short structural alignment length (shown in brown).
(DOC)

Table S3 **Intermodular angles for CCPs 18–20.** Tilt, twist and skew angles in degrees were determined as previously described [1], [2] using (for a reference *x*-axis) a vector between the principal axis of the inertia tensor (the *z*-axis) and the alpha-carbons of the conserved Trp1096 (CCP 18), Trp1157 (CCP 19) or Leu1223 (CCP 20), respectively, and with module boundaries defined as Cys-I i.e., Cys1048 (CCP 18), Cys1109 (CCP 19), or Cys1167 (CCP 20) and Cys-IV i.e., Cys1102 (CCP 18), Cys1163 (CCP 19), or Cys1228 (CCP 20), respectively.
(DOC)

Table S4 **Accessible surface area and interface buried surface area calculation.** The web server VADAR version 1.8 [1] was used and the surface area (SA) that was buried was calculated as: (SA Module 1+SA Module 2) - SA Bi-module. All units are in Å^2^. The linker length was defined as the number of residues between the C-terminal Cys of the preceding CCP module and the N-terminal Cys of the following CCP module. For CCP 18 (in CCP 18–CCP 19), boundaries were considered from one residue before the first Cys till three residues after the last Cys and for CCP 19 (in CCP 18–CCP 19 and CCP 19–CCP 20), boundaries were considered from three residues before the first Cys till one residue after the last Cys (and one residue before the first Cys till one residue after last Cys in CCP 19–CCP 20, and two residues before first Cys till one residue after last Cys in CCP 20). For bi-modules, boundaries were considered from one residue before the first Cys of Module 1 to one residue after the last Cys of Module 2.
(DOC)

## References

[1] Schmidt CQ, Herbert AP, Hocking HG, Uhrin D, Barlow PN (2008). Translational mini-review series on complement factor H: structural and functional correlations for factor H.. Clin Exp Immunol.

[2] Kirkitadze MD, Barlow PN (2001). Structure and flexibility of the multiple domain proteins that regulate complement activation.. Immunol Rev.

[3] Norman DG, Barlow PN, Baron M, Day AJ, Sim RB (1991). Three-dimensional structure of a complement control protein module in solution.. J Mol Biol.

[4] Ripoche J, Day AJ, Harris TJR, Sim RB (1988). The complete amino acid sequence of human complement factor H.. Biochem J.

[5] Pangburn MK, Schreiber RD, Muller-Eberhard HJ (1977). Human complement C3b inactivator: isolation, characterization, and demonstration of an absolute requirement for the serum protein beta1H for cleavage of C3b and C4b in solution.. J Exp Med.

[6] Weiler JM, Daha MR, Austen KF, Fearon DT (1976). Control of the amplification convertase of complement by the plasma protein beta1H.. Proc Natl Acad Sci U S A.

[7] Whaley K, Ruddy S (1976). Modulation of the alternative complement pathways by beta 1 H globulin.. J Exp Med.

[8] Meri S, Pangburn MK (1990). Discrimination between activators and nonactivators of the alternative pathway of complement: regulation via a sialic acid/polyanion binding site on factor H.. Proc Natl Acad Sci U S A.

[9] Meri S, Pangburn MK (1994). Regulation of alternative pathway complement activation by glycosaminoglycans: specificity of the polyanion binding site on factor H.. Biochem Biophys Res Commun.

[10] Pangburn MK (2000). Host recognition and target differentiation by factor H, a regulator of the alternative pathway of complement.. Immunopharmacology.

[11] Gordon DL, Kaufman RM, Blackmore TK, Kwong J, Lublin DM (1995). Identification of complement regulatory domains in human factor H.. J Immunol.

[12] Pangburn MK (2002). Cutting edge: localization of the host recognition functions of complement factor H at the carboxyl-terminal: implications for hemolytic uremic syndrome.. J Immunol.

[13] Schmidt CQ, Herbert AP, Kavanagh D, Gandy C, Fenton CJ (2008). A new map of glycosaminoglycan and C3b binding sites on factor H.. J Immunol.

[14] Sharma AK, Pangburn MK (1996). Identification of three physically and functionally distinct binding sites for C3b in human complement factor H by deletion mutagenesis.. Proc Natl Acad Sci U S A.

[15] Wu J, Wu YQ, Ricklin D, Janssen BJ, Lambris JD (2009). Structure of complement fragment C3b-factor H and implications for host protection by complement regulators.. Nat Immunol.

[16] Pechtl IC, Neely RK, Dryden DTF, Jones AC, Barlow PN (2011). Use of time-resolved FRET to validate crystal structure of complement regulatory complex between C3b and factor H (N terminus).. Protein Sci.

[17] Kajander T, Lehtinen MJ, Hyvarinen S, Bhattacharjee A, Leung E (2011). Dual interaction of factor H with C3d and glycosaminoglycans in host-nonhost discrimination by complement.. Proc Natl Acad Sci U S A.

[18] Morgan HP, Schmidt CQ, Guariento M, Blaum BS, Gillespie D (2011). Structural basis for engagement by complement factor H of C3b on a self surface.. Nat Struct Mol Biol.

[19] Blackmore TK, Hellwage J, Sadlon TA, Higgs N, Zipfel PF (1998). Identification of the second heparin-binding domain in human complement factor H.. J Immunol.

[20] Blackmore TK, Sadlon TA, Ward HM, Lublin DM, Gordon DL (1996). Identification of a heparin binding domain in the seventh short consensus repeat of complement factor H.. J Immunol.

[21] Herbert AP, Deakin JA, Schmidt CQ, Blaum BS, Egan C (2007). Structure shows that a glycosaminoglycan and protein recognition site in factor H is perturbed by age-related macular degeneration-linked single nucleotide polymorphism.. J Biol Chem.

[22] Morgan HP, Jiang J, Herbert AP, Kavanagh D, Uhrin D (2011). Crystallographic determination of the disease-associated T1184R variant of complement regulator factor H.. Acta Crystallogr D Biol Crystallogr.

[23] Pangburn MK, Atkinson MA, Meri S (1991). Localization of the heparin-binding site on complement factor H.. J Biol Chem.

[24] Prosser BE, Johnson S, Roversi P, Herbert AP, Blaum BS (2007). Structural basis for complement factor H linked age-related macular degeneration.. J Exp Med.

[25] Aslam M, Perkins SJ (2001). Folded-back solution structure of monomeric factor H of human complement by synchrotron X-ray and neutron scattering, analytical ultracentrifugation and constrained molecular modelling.. J Mol Biol.

[26] Fernando AN, Furtado PB, Clark SJ, Gilbert HE, Day AJ (2007). Associative and structural properties of the region of complement factor H encompassing the Tyr402His disease-related polymorphism and its interactions with heparin.. J Mol Biol.

[27] Okemefuna AI, Gilbert HE, Griggs KM, Ormsby RJ, Gordon DL (2008). The regulatory SCR-1/5 and cell surface-binding SCR-16/20 fragments of factor H reveal partially folded-back solution structures and different self-associative properties.. J Mol Biol.

[28] Oppermann M, Manuelian T, Jozsi M, Brandt E, Jokiranta TS (2006). The C-terminus of complement regulator Factor H mediates target recognition: evidence for a compact conformation of the native protein.. Clin Exp Immunol.

[29] Schmidt CQ, Herbert AP, Mertens HDT, Guariento M, Soares DC (2010). The Central Portion of Factor H (Modules 10–15) Is Compact and Contains a Structurally Deviant CCP Module.. J Mol Biol.

[30] de Cordoba SR, de Jorge EG (2008). Translational mini-review series on complement factor H: genetics and disease associations of human complement factor H.. Clin Exp Immunol.

[31] Boon CJ, Klevering BJ, Hoyng CB, Zonneveld-Vrieling MN, Nabuurs SB (2008). Basal laminar drusen caused by compound heterozygous variants in the CFH gene.. Am J Hum Genet.

[32] Perkins SJ, Goodship TH (2002). Molecular modelling of the C-terminal domains of factor H of human complement: a correlation between haemolytic uraemic syndrome and a predicted heparin binding site.. J Mol Biol.

[33] Raychaudhuri S, Iartchouk O, Chin K, Tan PL, Tai AK (2011). A rare penetrant mutation in CFH confers high risk of age-related macular degeneration.. Nat Genet.

[34] Richards A, Buddles MR, Donne RL, Kaplan BS, Kirk E (2001). Factor H mutations in hemolytic uremic syndrome cluster in exons 18–20, a domain important for host cell recognition.. Am J Hum Genet.

[35] Saunders RE, Goodship TH, Zipfel PF, Perkins SJ (2006). An interactive web database of factor H-associated hemolytic uremic syndrome mutations: insights into the structural consequences of disease-associated mutations.. Hum Mutat.

[36] Guigonis V, Fremeaux-Bacchi V, Giraudier S, Favier R, Borderie D (2005). Late-onset thrombocytic microangiopathy caused by cblC disease: association with a factor H mutation.. Am J Kidney Dis.

[37] Herbert AP, Uhrin D, Lyon M, Pangburn MK, Barlow PN (2006). Disease-associated sequence variations congregate in a polyanion recognition patch on human factor H revealed in three-dimensional structure.. J Biol Chem.

[38] Potterton E, Briggs P, Turkenburg M, Dodson E (2003). A graphical user interface to the CCP4 program suite.. Acta Crystallogr D Biol Crystallogr.

[39] Evans P (2006). Scaling and assessment of data quality.. Acta Crystallogr D Biol Crystallogr.

[40] McCoy AJ, Grosse-Kunstleve RW, Adams PD, Winn MD, Storoni LC (2007). Phaser crystallographic software.. J Appl Crystallogr.

[41] Murshudov GN, Vagin AA, Dodson EJ (1997). Refinement of macromolecular structures by the maximum-likelihood method.. Acta Crystallogr D Biol Crystallogr.

[42] Terwilliger TC, Grosse-Kunstleve RW, Afonine PV, Moriarty NW, Zwart PH (2008). Iterative model building, structure refinement and density modification with the PHENIX AutoBuild wizard.. Acta Crystallogr D Biol Crystallogr.

[43] Emsley P, Cowtan K (2004). Coot: model-building tools for molecular graphics.. Acta Crystallogr D-Biol Crystallogr.

[44] Davis IW, Leaver-Fay A, Chen VB, Block JN, Kapral GJ (2007). MolProbity: all-atom contacts and structure validation for proteins and nucleic acids.. Nucleic Acids Res.

[45] Maleknia SD, Ralston CY, Brenowitz MD, Downard KM, Chance MR (2001). Determination of macromolecular folding and structure by synchrotron x-ray radiolysis techniques.. Anal Biochem.

[46] Konarev PV, Volkov VV, Sokolova AV, Koch MHJ, Svergun DI (2003). PRIMUS: a Windows PC-based system for small-angle scattering data analysis.. J Appl Crystallogr.

[47] Svergun D, Barberato C, Koch MHJ (1995). CRYSOL - a Program to Evaluate X-ray Solution Scattering of Biological Macromolecules from Atomic Coordinates.. J Appl Crystallogr.

[48] Franke D, Svergun DI (2009). DAMMIF, a program for rapid ab-initio shape determination in small-angle scattering.. J Appl Crystallogr.

[49] Volkov VV, Svergun DI (2003). Uniqueness of ab initio shape determination in small-angle scattering.. J Appl Crystallogr.

[50] Kozin MB, Svergun DI (2001). Automated matching of high- and low-resolution structural models.. J Appl Crystallogr.

[51] Petoukhov MV, Svergun DI (2005). Global rigid body modeling of macromolecular complexes against small-angle scattering data.. Biophys J.

[52] Bernado P, Mylonas E, Petoukhov MV, Blackledge M, Svergun DI (2007). Structural characterization of flexible proteins using small-angle X-ray scattering.. J Am Chem Soc.

[53] Bhattacharjee A, Lehtinen MJ, Kajander T, Goldman A, Jokiranta TS (2010). Both domain 19 and domain 20 of factor H are involved in binding to complement C3b and C3d.. Mol Immunol.

[54] Jokiranta TS, Jaakola VP, Lehtinen MJ, Parepalo M, Meri S (2006). Structure of complement factor H carboxyl-terminus reveals molecular basis of atypical haemolytic uremic syndrome.. Embo J.

[55] Soares DC, Gerloff DL, Syme NR, Coulson AF, Parkinson J (2005). Large-scale modelling as a route to multiple surface comparisons of the CCP module family.. Protein Eng Des Sel.

[56] Kirkitadze MD, Henderson C, Price NC, Kelly SM, Mullin NP (1999). Central modules of the vaccinia virus complement control protein are not in extensive contact.. Biochem J.

[57] Kirkitadze MD, Krych M, Uhrin D, Dryden DT, Smith BO (1999). Independently melting modules and highly structured intermodular junctions within complement receptor type 1.. Biochemistry.

[58] Soares DC, Barlow PN, Morikis D, Lambris JD (2005). Complement control protein modules in the regulators of complement activation.. Structural Biology of the Complement System.

[59] Ferreira VP, Herbert AP, Cortes C, McKee KA, Blaum BS (2009). The Binding of Factor H to a Complex of Physiological Polyanions and C3b on Cells Is Impaired in Atypical Hemolytic Uremic Syndrome.. J Immunol.

[60] Lehtinen MJ, Rops AL, Isenman DE, van der Vlag J, Jokiranta TS (2009). Mutations of factor H impair regulation of surface-bound C3b by three mechanisms in atypical hemolytic uremic syndrome.. J Biol Chem.

[61] Okemefuna AI, Nan R, Miller A, Gor J, Perkins SJ (2010). Complement factor H binds at two independent sites to C-reactive protein in acute phase concentrations.. J Biol Chem.

[62] Guinier A (1939). La diffraction des rayons X aux tres petits angles; application a l'etude de phenomenes ultramicroscopiques.. Ann Phys (Paris).

[63] Svergun DI (1992). Determination of the regularization parameter in indirect-transform methods using perceptual criteria.. J Appl Crystallogr.

[64] Baker NA, Sept D, Joseph S, Holst MJ, McCammon JA (2001). Electrostatics of nanosystems: application to microtubules and the ribosome.. Proc Natl Acad Sci U S A.

