# Supplementary material for: Structural Analysis of the C-Terminal Region (Modules 18–20) of Complement Regulator Factor H (FH)
Source: PLoS One. 2012 Feb 28;7(2):e32187. doi: 10.1371/journal.pone.0032187 (PMC3289644; doi:10.1371/journal.pone.0032187)
Supplement: Figure S1 — Fit of FH18–20 rigid body models refined against the SAXS data using the program CORAL. Fits are shown for rigid body models refining only CCP 18 (CCPs 19–20 fixed) or CCP 20 (CCPs 18–19 fixed), as solid blue and dashed red lines, respectively. (PPT) [file pone.0032187.s001.ppt]

## Slide 1
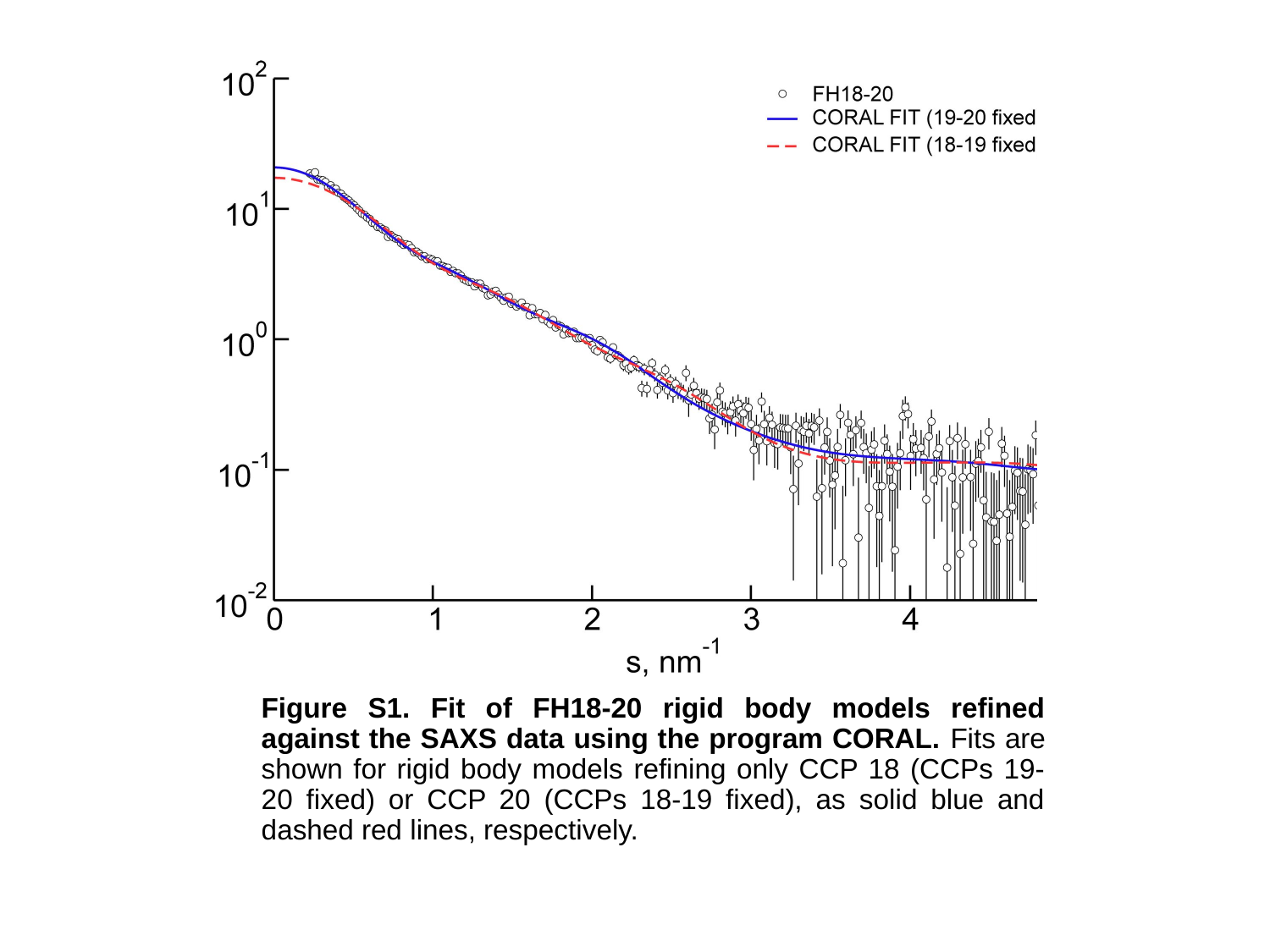

# Figure S1. Fit of FH18-20 rigid body models refined against the SAXS data using the program CORAL. Fits are shown for rigid body models refining only CCP 18 (CCPs 19-20 fixed) or CCP 20 (CCPs 18-19 fixed), as solid blue and dashed red lines, respectively.
