# Supplementary material for: Structural Analysis of the C-Terminal Region (Modules 18–20) of Complement Regulator Factor H (FH)
Source: PLoS One. 2012 Feb 28;7(2):e32187. doi: 10.1371/journal.pone.0032187 (PMC3289644; doi:10.1371/journal.pone.0032187)
Supplement: Table S1 — Comparison of the discrepancies (χ) for the DAMMIF, CORAL and EOM SAXS-derived analyses. (DOC) [file pone.0032187.s002.doc]

| **Model** | **CORAL** | **DAMMIF** | **EOM** |
| --- | --- | --- | --- |
| FH19-20 | - | 1.4 | 1.1 |
| FH18-20 | 1.3 | 1.3 | 1.2 |
| FH18-19 fixed | 2.1 | - | 2.4 |
| FH19-20 fixed | 1.4 | - | 1.3 |

**Table S1.** **Comparison of the discrepancies (χ) for the DAMMIF, CORAL and EOM SAXS-derived analyses.**
