# Supplementary material for: Structural Analysis of the C-Terminal Region (Modules 18–20) of Complement Regulator Factor H (FH)
Source: PLoS One. 2012 Feb 28;7(2):e32187. doi: 10.1371/journal.pone.0032187 (PMC3289644; doi:10.1371/journal.pone.0032187)
Supplement: Table S2 — Pair-wise CCP module structural comparisons of FH18, FH19 and FH20. Comparison of CCP module structures of CCP 18, CCP 19 and CCP 20 versus all other individual CCPs of known structure within the complement system based upon alpha-carbon RMSD values using the structural alignment program Combinatorial Extension [22]. For each CCP, inclusive module boundaries were one residue before Cys-I and the third residue after Cys-IV. In cases where structures have been solved by both NMR and X-ray diffraction, the higher resolution X-ray structure was used for comparison. Where both liganded and unliganded structures were available, the highest resolution unliganded X-ray or NMR structure was used. A few residues were missing in the crystal structure of C1r CCP 2, and hence in this case, the structure with the most determined residues was employed for both modules. Colour key used in table: Blue: 0–1.99 Å; Green: 2.00–2.99 Å; Red: 3.00–3.99 Å; Brown: Alignment lengths <40 amino acids. Abbreviations used in Table: C4BPα = C4b-binding protein α-chain; CR = complement receptor; CRRY = rat Complement receptor 1-related protein Y; DAF = decay-accelerating factor; FB = factor B; FH = factor H; MASP1/2 = mannan-binding lectin-associated serine proteases 1/2; MCP = membrane cofactor protein; VCP = Vaccinia virus complement control protein. Some residues were not present (solved) in the electron density map for the C1r CCP 2 module crystal structure, and this explains the short structural alignment length (shown in brown). (DOC) [file pone.0032187.s003.doc]

| **Protein; module; PDB ID** | **RMSD in Å (alignment length, gaps included)** | | |
| --- | --- | --- | --- |
|  | **FH18 (3SW0)** | **FH19 (3SW0)** | **FH20 (3SW0)** |
| C1r; CCP 1; (1GPZ) | 1.77 (58) | 1.80 (58) | 2.72 (60) |
| C1r; CCP 2; (1GPZ) | 1.85 (35) | 2.32 (35) | 4.60 (36) |
| C1s; CCP 2; (1ELV) | 1.97 (57) | 2.03 (57) | 3.49 (70) |
| C2; CCP1; (3ERB) | 2.31 (49) | 2.72 (49) | 3.61 (61) |
| C2; CCP2; (3ERB) | 2.00 (58) | 1.99 (58) | 2.13 (56) |
| C2; CCP3; (3ERB) | 1.66 (56) | 1.70 (56) | 3.32 (59) |
| C4BPα; CCP 1; (2A55) | 2.52 (59) | 2.57 (59) | 3.18 (59) |
| C4BPα; CCP 2; (2A55) | 1.98 (56) | 2.23 (57) | 3.33 (61) |
| C7; CCP 1; (Clark *et al*., unpublished) | 2.12 (57) | 2.20 (57) | 2.64 (55) |
| C7; CCP2; (Clark *et al*., unpublished) | 2.31 (57) | 2.46 (57) | 2.87 (56) |
| CR1; CCP 15; (1GKN) | 2.04 (57) | 2.33 (57) | 3.17 (57) |
| CR1; CCP 16; (1GKN) | 1.90 (57) | 2.25 (57) | 2.57 (55) |
| CR1; CCP 17; (1GKG) | 1.70 (57) | 1.98 (57) | 3.61 (73) |
| CR2; CCP 1; (1LY2) | 1.16 (58) | 1.32 (58) | 3.01 (61) |
| CR2; CCP 2; (1LY2) | 1.28 (57) | 1.55 (57) | 2.27 (59) |
| CRRY; CCP 1; (2XRB) | 2.75 (58) | 2.65 (58) | 2.84 (56) |
| CRRY; CCP 2; (2XRB) | 1.92 (57) | 2.15 (57) | 2.95 (61) |
| CRRY; CCP 3; (2XRB) | 1.45 (59) | 1.50 (59) | 2.43 (57) |
| CRRY; CCP 4; (2XRB) | 1.25 (57) | 1.39 (57) | 2.45 (56) |
| DAF; CCP 1; (1OK3) | 2.30 (59) | 2.27 (59) | 2.79 (59) |
| DAF; CCP 2 (1OK3) | 2.39 (56) | 2.30 (59) | 3.02 (60) |
| DAF; CCP 3; (1H03) | 1.82 (57) | 2.22 (57) | 3.01 (61) |
| DAF; CCP4; (1H03) | 1.17 (57) | 1.11 (57) | 1.91 (48) |
| FB; CCP 1; (2OK5) | 2.43 (47) | 3.58 (76) | 4.36 (63) |
| FB; CCP 2; (2OK5) | 1.76 (58) | 1.74 (58) | 2.12 (56) |
| FB; CCP 3; (2OK5) | 1.76 (56) | 1.81 (56) | 3.14 (60) |
| FH; CCP 1; (2RLP) | 2.41 (57) | 2.31 (57) | 3.12 (57) |
| FH; CCP 2; (2RLQ) | 2.16 (55) | 2.27 (54) | 3.23 (56) |
| FH; CCP 3; (2RLQ) | 1.84 (57) | 1.82 (57) | 2.98 (59) |
| FH; CCP 4; (2WII) | 1.81 (57) | 1.79 (57) | 2.30 (55) |
| FH; CCP 5; (not deposited) | 2.21 (53) | 2.25 (53) | 2.41 (56) |
| FH; CCP 6; (2UWN) | 2.39 (56) | 2.45 (56) | 2.75 (58) |
| FH; CCP 7; (2UWN) | 2.98 (56) | 2.94 (57) | 3.05 (56) |
| FH; CCP 8; (2UWN) | 1.88 (51) | 1.86 (51) | 2.67 (56) |
| FH; CCP 12; (2KMS) | 1.45 (58) | 1.46 (58) | 2.68 (56) |
| FH; CCP 13; (2KMS) | 3.10 (48) | 3.19 (55) | 2.88 (64) |
| FH; CCP 15; (1HFH) | 2.14 (59) | 2.14 (58) | 2.71 (59) |
| FH; CCP 16; (1HFH) | 2.10 (54) | 2.11 (54) | 2.98 (54) |
| FH; CCP 18; (3SW0) | - | 1.08 (59) | 2.32 (57) |
| FH; CCP 19; (2G7I) | 1.16 (59) | 0.40 (59) | 2.26 (57) |
| FH; CCP 19; (3SW0) | 1.08 (59) | - | 2.24 (57) |
| FH; CCP 20; (2G7I) | 2.17 (57) | 2.23 (57) | 1.22 (65) |
| FH; CCP 20; (3SW0) | 2.32 (57) | 2.24 (57) | - |
| MASP1; CCP 1; (3GOV) | 1.85 (58) | 1.81 (58) | 3.00 (59) |
| MASP1; CCP 2; (3GOV) | 1.73 (57) | 1.92 (57) | 3.07 (55) |
| MASP2; CCP 1; (1ZJK) | 1.73 (58) | 1.62 (58) | 2.77 (59) |
| MASP2; CCP 2; (1ZJK) | 1.89 (59) | 1.87 (58) | 2.88 (58) |
| MCP; CCP 1; (1CKL) | 2.10 (55) | 1.92 (55) | 2.48 (58) |
| MCP; CCP 2; (1CKL) | 2.43 (58) | 2.73 (58) | 3.27 (59) |
| MCP; CCP 3; (3O8E) | 1.49 (59) | 1.31 (59) | 2.62 (57) |
| MCP; CCP 4; (3O8E) | 1.65 (58) | 1.66 (54) | 3.05 (65) |
| VCP; CCP 1; (1G40) | 2.22 (59) | 2.49 (59) | 2.67 (62) |
| VCP; CCP 2; (1G40) | 2.00 (51) | 2.03 (56) | 4.40 (58) |
| VCP; CCP 3; (1G40) | 2.01 (57) | 2.10 (57) | 2.52 (56) |
| VCP; CCP 4; (1G40) | 1.59 (56) | 1.77 (56) | 2.35 (55) |

**Table S2. Pair-wise CCP module structural comparisons of FH18, FH19 and FH20.** Comparison of CCP module structures of CCP 18, CCP 19 and CCP 20 versus all other individual CCPs of known structure within the complement system based upon alpha-carbon RMSD values using the structural alignment program Combinatorial Extension . For each CCP, inclusive module boundaries were one residue before Cys-I and the third residue after Cys-IV. In cases where structures have been solved by both NMR and X-ray diffraction, the higher resolution X-ray structure was used for comparison. Where both liganded and unliganded structures were available, the highest resolution unliganded X-ray or NMR structure was used. A few residues were missing in the crystal structure of C1r CCP 2, and hence in this case, the structure with the most determined residues was employed for both modules. Colour key used in table: Blue: 0 - 1.99 Å; Green: 2.00 – 2.99 Å; Red: 3.00 –3.99 Å; Brown: Alignment lengths < 40 amino acids. Abbreviations used in Table: C4BPα = C4b-binding protein α-chain; CR = complement receptor; CRRY = rat Complement receptor 1-related protein Y; DAF = decay-accelerating factor; FB = factor B; FH = factor H; MASP1 / 2 = mannan-binding lectin-associated serine proteases 1 / 2; MCP = membrane cofactor protein; VCP = Vaccinia virus complement control protein. Some residues were not present (solved) in the electron density map for the C1r CCP 2 module crystal structure, and this explains the short structural alignment length (shown in brown).

**References**

1. Budayova-Spano M, Lacroix M, Thielens NM, Arlaud GJ, Fontecilla-Camps JC, et al. (2002) The crystal structure of the zymogen catalytic domain of complement protease C1r reveals that a disruptive mechanical stress is required to trigger activation of the C1 complex. Embo J 21: 231-239.

2. Gaboriaud C, Rossi V, Bally I, Arlaud GJ, Fontecilla-Camps JC (2000) Crystal structure of the catalytic domain of human complement c1s: a serine protease with a handle. Embo J 19: 1755-1765.

3. Krishnan V, Xu Y, Macon K, Volanakis JE, Narayana SV (2009) The structure of C2b, a fragment of complement component C2 produced during C3 convertase formation. Acta Crystallogr D Biol Crystallogr 65: 266-274.

4. Jenkins HT, Mark L, Ball G, Persson J, Lindahl G, et al. (2006) Human C4b-binding protein, structural basis for interaction with streptococcal M protein, a major bacterial virulence factor. J Biol Chem 281: 3690-3697.

5. Smith BO, Mallin RL, Krych-Goldberg M, Wang X, Hauhart RE, et al. (2002) Structure of the C3b binding site of CR1 (CD35), the immune adherence receptor. Cell 108: 769-780.

6. Prota AE, Sage DR, Stehle T, Fingeroth JD (2002) The crystal structure of human CD21: Implications for Epstein-Barr virus and C3d binding. Proc Natl Acad Sci U S A 99: 10641-10646.

7. Roversi P, Johnson S, Caesar JJ, McLean F, Leath KJ, et al. (2011) Structures of the rat complement regulator CrrY. Acta Crystallogr Sect F Struct Biol Cryst Commun 67: 739-743.

8. Lukacik P, Roversi P, White J, Esser D, Smith GP, et al. (2004) Complement regulation at the molecular level: the structure of decay-accelerating factor. Proc Natl Acad Sci U S A 101: 1279-1284.

9. Williams P, Chaudhry Y, Goodfellow IG, Billington J, Powell R, et al. (2003) Mapping CD55 function. The structure of two pathogen-binding domains at 1.7 A. J Biol Chem 278: 10691-10696.

10. Milder FJ, Gomes L, Schouten A, Janssen BJ, Huizinga EG, et al. (2007) Factor B structure provides insights into activation of the central protease of the complement system. Nat Struct Mol Biol 14: 224-228.

11. Hocking HG, Herbert AP, Kavanagh D, Soares DC, Ferreira VP, et al. (2008) Structure of the N-terminal region of complement factor H and conformational implications of disease-linked sequence variations. J Biol Chem 283: 9475-9487.

12. Wu J, Wu YQ, Ricklin D, Janssen BJ, Lambris JD, et al. (2009) Structure of complement fragment C3b-factor H and implications for host protection by complement regulators. Nat Immunol 10: 728-733.

13. Prosser BE, Johnson S, Roversi P, Herbert AP, Blaum BS, et al. (2007) Structural basis for complement factor H linked age-related macular degeneration. J Exp Med 204: 2277-2283.

14. Schmidt CQ, Herbert AP, Mertens HDT, Guariento M, Soares DC, et al. (2010) The Central Portion of Factor H (Modules 10-15) Is Compact and Contains a Structurally Deviant CCP Module. J Mol Biol 395: 105-122.

15. Barlow PN, Steinkasserer A, Norman DG, Kieffer B, Wiles AP, et al. (1993) Solution structure of a pair of complement modules by nuclear magnetic resonance. J Mol Biol 232: 268-284.

16. Jokiranta TS, Jaakola VP, Lehtinen MJ, Parepalo M, Meri S, et al. (2006) Structure of complement factor H carboxyl-terminus reveals molecular basis of atypical haemolytic uremic syndrome. Embo J 25: 1784-1794.

17. Dobo J, Harmat V, Beinrohr L, Sebestyen E, Zavodszky P, et al. (2009) MASP-1, a promiscuous complement protease: structure of its catalytic region reveals the basis of its broad specificity. J Immunol 183: 1207-1214.

18. Gal P, Harmat V, Kocsis A, Bian T, Barna L, et al. (2005) A true autoactivating enzyme. Structural insight into mannose-binding lectin-associated serine protease-2 activations. J Biol Chem 280: 33435-33444.

19. Casasnovas JM, Larvie M, Stehle T (1999) Crystal structure of two CD46 domains reveals an extended measles virus-binding surface. Embo J 18: 2911-2922.

20. Persson BD, Schmitz NB, Santiago C, Zocher G, Larvie M, et al. (2010) Structure of the extracellular portion of CD46 provides insights into its interactions with complement proteins and pathogens. PLoS Pathog 6: e1001122.

21. Murthy KH, Smith SA, Ganesh VK, Judge KW, Mullin N, et al. (2001) Crystal structure of a complement control protein that regulates both pathways of complement activation and binds heparan sulfate proteoglycans. Cell 104: 301-311.

22. Shindyalov IN, Bourne PE (1998) Protein structure alignment by incremental combinatorial extension (CE) of the optimal path. Protein Eng 11: 739-747.
