# Supplementary material for: Structural Analysis of the C-Terminal Region (Modules 18–20) of Complement Regulator Factor H (FH)
Source: PLoS One. 2012 Feb 28;7(2):e32187. doi: 10.1371/journal.pone.0032187 (PMC3289644; doi:10.1371/journal.pone.0032187)
Supplement: Table S3 — Intermodular angles for CCPs 18–20. Tilt, twist and skew angles in degrees were determined as previously described [1], [2] using (for a reference x-axis) a vector between the principal axis of the inertia tensor (the z-axis) and the alpha-carbons of the conserved Trp1096 (CCP 18), Trp1157 (CCP 19) or Leu1223 (CCP 20), respectively, and with module boundaries defined as Cys-I i.e., Cys1048 (CCP 18), Cys1109 (CCP 19), or Cys1167 (CCP 20) and Cys-IV i.e., Cys1102 (CCP 18), Cys1163 (CCP 19), or Cys1228 (CCP 20), respectively. (DOC) [file pone.0032187.s004.doc]

| **PDB ID: 3SW0** | **Tilt** | **Twist** | **Skew** |
| --- | --- | --- | --- |
| CCP 18-CCP 19 | 121.97 | 33.74 | 141.72 |
| CCP 19-CCP 20 | 32.71 | 59.24 | 130.73 |
| CCP 18-CCP 20 | 151.18 | 132.64 | 141.03 |

**Table S3. Intermodular angles for CCPs 18-20.** Tilt, twist and skew angles in degrees were determined as previously described [1,2] using (for a reference *x*-axis) a vector between the principal axis of the inertia tensor (the *z*-axis) and the alpha-carbons of the conserved Trp1096 (CCP 18), Trp1157 (CCP 19) or Leu1223 (CCP 20), respectively, and with module boundaries defined as Cys-I i.e., Cys1048 (CCP 18), Cys1109 (CCP 19), or Cys1167 (CCP 20) and Cys-IV i.e., Cys1102 (CCP 18), Cys1163 (CCP 19), or Cys1228 (CCP 20), respectively.

**References**

1. Barlow PN, Steinkasserer A, Norman DG, Kieffer B, Wiles AP, et al. (1993) Solution structure of a pair of complement modules by nuclear magnetic resonance. J Mol Biol 232: 268-284.

2. Soares DC, Barlow PN (2005) Complement control protein modules in the regulators of complement activation. In: Morikis D, Lambris JD, editors. Structural Biology of the Complement System. Boca Raton: CRC Press, Taylor & Francis Group. pp. 19-62.
