# Supplementary material for: Structural Analysis of the C-Terminal Region (Modules 18–20) of Complement Regulator Factor H (FH)
Source: PLoS One. 2012 Feb 28;7(2):e32187. doi: 10.1371/journal.pone.0032187 (PMC3289644; doi:10.1371/journal.pone.0032187)
Supplement: Table S4 — Accessible surface area and interface buried surface area calculation. The web server VADAR version 1.8 [1] was used and the surface area (SA) that was buried was calculated as: (SA Module 1+SA Module 2) - SA Bi-module. All units are in Å2. The linker length was defined as the number of residues between the C-terminal Cys of the preceding CCP module and the N-terminal Cys of the following CCP module. For CCP 18 (in CCP 18–CCP 19), boundaries were considered from one residue before the first Cys till three residues after the last Cys and for CCP 19 (in CCP 18–CCP 19 and CCP 19–CCP 20), boundaries were considered from three residues before the first Cys till one residue after the last Cys (and one residue before the first Cys till one residue after last Cys in CCP 19–CCP 20, and two residues before first Cys till one residue after last Cys in CCP 20). For bi-modules, boundaries were considered from one residue before the first Cys of Module 1 to one residue after the last Cys of Module 2. (DOC) [file pone.0032187.s005.doc]

| **CCP Module Fragment** | **Module 1** | **Module 2** | **Bi-module** | **Buried** |
| --- | --- | --- | --- | --- |
| CCP 18-CCP 19 | 4129.4 (CCP 18) | 4123.1 (CCP 19) | 7845.9 (CCP 18-19) | 406.4 |
| CCP 19-CCP 20 | 4029.3 (CCP 19) | 4840.5 (CCP 20) | 8170.4 (CCP 19-20) | 699.4 |

**Table S4. Accessible surface area and interface buried surface area calculation.** The web server VADAR version 1.8 [1] was used and the surface area (SA) that was buried was calculated as: (SA Module 1 + SA Module 2) - SA Bi-module. All units are in Å^2^. The linker length was defined as the number of residues between the C-terminal Cys of the preceding CCP module and the N-terminal Cys of the following CCP module. For CCP 18 (in CCP 18-CCP 19), boundaries were considered from one residue before the first Cys till three residues after the last Cys and for CCP 19 (in CCP 18-CCP 19 and CCP 19-CCP 20), boundaries were considered from three residues before the first Cys till one residue after the last Cys (and one residue before the first Cys till one residue after last Cys in CCP 19-CCP 20, and two residues before first Cys till one residue after last Cys in CCP 20). For bi-modules, boundaries were considered from one residue before the first Cys of Module 1 to one residue after the last Cys of Module 2.

**References**

1. Willard L, Ranjan A, Zhang H, Monzavi H, Boyko RF, et al. (2003) VADAR: a web server for quantitative evaluation of protein structure quality. Nucleic Acids Res 31: 3316-3319.
